# Food Choices and Hypertension Among Rural Thais: Evidence From a Discrete Choice Experiment

**DOI:** 10.3389/ijph.2022.1604850

**Published:** 2022-07-15

**Authors:** Pimbucha Rusmevichientong, Helen Nguyen, Celina Morales, Jessica Jaynes, Michele M. Wood

**Affiliations:** ^1^ Department of Public Health, California State University Fullerton, Fullerton, CA, United States; ^2^ School of Public Health, San Diego State University, San Diego, CA, United States; ^3^ Department of Population Health and Disease Prevention, University of California Irvine, Irvine, CA, United States; ^4^ Department of Mathematics, California State University Fullerton, Fullerton, CA, United States

**Keywords:** hypertension, food choices, rural Thailand, discrete choice experiment (DCE), salt

## Abstract

**Objective:** The rural northern region of Thailand exhibits the highest rate of hypertension. This study explored hypertensive-related food choices between normotensive and hypertensive people residing in rural northern Thailand to determine which food attributes influence their choices.

**Methods:** The study conducted a discrete choice experiment (DCE) survey among Thai adults residing in rural northern Thailand (*n* = 403) to estimate the relative importance of four food attributes, including food preparation, price, taste, and amount of salt. A mixed logit model was used to analyze the data from the DCE.

**Results:** The first and second most important attributes in both hypertensive and normotensive groups were the amount of salt and food preparation at home, respectively, followed by price and taste. Specifically, the normotensive group was more attentive to the amount of salt in their food than their hypertensive counterparts.

**Conclusion:** Intervention programs in rural communities may benefit from focusing their attention on embracing low-salt cultural foods and providing guidance on how to add flavor without additional salt or reduce high sodium seasonings without losing flavor when cooking.

## Introduction

Hypertension, also known as high blood pressure, is a major public health concern and one of the most contributing risk factors for cardiovascular diseases (CVDs). About half of the world’s CVD burden occurs in the Asia Pacific region [1], and 30% of adults in Southeast Asia have elevated blood pressure [2]. As of 2019, one out of four Thai adults have hypertension, equating to 13.2 million people, and only 3.9 million have their blood pressure under control; more than 50,000 deaths annually are attributed to hypertension, where CVDs account for a quarter of all deaths [3]. A study on CVD patients in the northern region of Thailand found that 84% of hypertensive individuals did not know they had hypertension, and 73% of pre-hypertensive individuals did not think they were at risk [4]. Particularly, evidence indicates that the northern region of Thailand exhibits the highest rate of hypertension (33%) [3], where people living in this region consume a considerable amount of salt (3,044 mg/day) compared to those living in other regions [5].

Sodium consumption associated with hypertension in the Thai population has been extensively studied [6–8]. Thai adults consume, on average, 10.8 g of salt a day (4,320 mg of sodium), which is more than double the WHO recommended amount of daily salt (5 g/day ∼2,000 mg of sodium) [9]. Popular food choices such as one-plate meals, meat products, snacks, beverages, and fast food [10] are common sources of high sodium intake among Thai people. According to Nielsen’s global eating trend survey [11], Asia-Pacific people are avid out-of-home diners. In fact, 22% of Thai respondents, compared to 9% of global respondents, reported eating away from home at least once a day [12]. Moreover, 38% of Thai respondents who eat away from home reported eating street foods [12], which are a popular choice for out-of-home dining in Thailand and are commonly high in sodium [13].

### Theoretical Framework and Hypotheses

Although the pathophysiological link between hypertension and sodium intake has been debated [14, 15], the strong association between these two has been widely recognized and supported in the literature [16]. A reduction in sodium intake can have a favorable effect on the cardiovascular system, inducing a reduction in blood pressure, particularly in hypertensive patients [14]. Furthermore, food choices can be an indicator of nutrient intake, leading to overall health status [17]. Thus, understanding individuals’ food choices and encouraging behavioral changes to promote healthier decisions can reduce the burden of hypertension-related complications [18]. However, the theoretical framework and methodologies in the literature do not account for the complexity in understanding the factors that underpin individual food choices.

The theory of planned behavior (TPB) [19], a psychological theory to predict an individual’s intention to engage in a specific behavior, has shown that food choices are complex and involve a combination of three fundamental components: 1) attitude, an overall favorable or unfavorable evaluation of the behavior; 2) personal and social norm, an individual’s sense of self obligation and accepted standard of behavior in specific situations, respectively; and 3) perceived behavioral control (PBC), a perception of the ease or difficulty of enacting the behavior [5, 20]. Mørk et al. [5] applied the TPB to food choices and found that positive salt-related attitudes (i.e., awareness of hypertension consequences), strong personal norms toward salt reduction, together with knowledge of salt-related diseases and diets can exert the strongest influence on the willingness to purchase food products low in salt.

While several studies have examined individual’s salt-related dietary knowledge, attitudes, and practices [6, 21–23], the results have been inconclusive. To complement the literature on TBP related to food choice behaviors, this study investigates individuals’ intention of selecting hypertension-related food between normotensive and hypertensive people in rural Thailand by constructing a choice-based survey. As food choices involve a complexity of conscious trade-offs such as money, nutrition, taste, etc. [24], this study utilizes a discrete choice experiment (DCE), which closely resembles real-world decision-making processes [25], to answer the following hypotheses (H1-H3):

H1: In foods that contain high salt, people with hypertension will make different trade-offs in their food choices than normotensive people.
H2: The relative importance of the attribute, amount of salt, will be lower among people with hypertension than normotensive people.
H3: People with hypertension will have different food choices than normotensive people.

## Methods

### Discrete Choice Experiment Design

In a DCE, subjects are presented with several questions known as choice sets, made up of several options that are constructed from different combinations of attributes [26]. Subjects are asked to select a single option. While existing survey methods such as rating, ranking, or focus group discussions, have been widely used to assess the attributes that influence food choices and health status [11, 12, 27, 28], they are unable to assess the interaction effect between two attributes. With a DCE, the interactions between attributes, and the relative importance of attributes can be identified and quantified [29], which captures the complexity of the trade-offs in decision making.

The design of the DCE refers to the selection of different combinations of attributes and levels to form the corresponding options and choice sets. It is critical in determining which attributes and interactions can be estimated. This study used an experimental design technique known as a block factorial design (BFD), which was utilized to design the DCEs in Rusmevichientong et al. 2021, 2020 [30, 31], and is based on the foundational work of Jaynes et al. 2016 [32].

The relationship between BFDs and DCEs is as follows. First, the number of blocks in a BFD refers to the number of choice sets. Second, the size of the blocks in a BFD refers to the number of options within each choice set. To construct a DCE using a BFD, start with either a full or fractional factorial block design, such that each block represents a choice set, and the number of combinations in the blocks represent the number of options within each choice set [30]. A couple of advantages of using a BFD for a DCE include 1) potentially reducing the number of choice sets while still answering the research question, and 2) estimating all individual effects and some interactions between two attributes.

This study considers four attributes denoted as A, B, C, and D, each with two levels. A full factorial design for four two-level attributes consists of 16 (2^4^) possible combinations, which are divided into four blocks, each with four combinations [33], representing a DCE with four choice sets, each with four options. With this design, all four attributes (A, B, C, D) plus five interactions between two attributes (AC, AD, BC, BD, CD) can be estimated unbiasedly.

**FIGURE 1:**
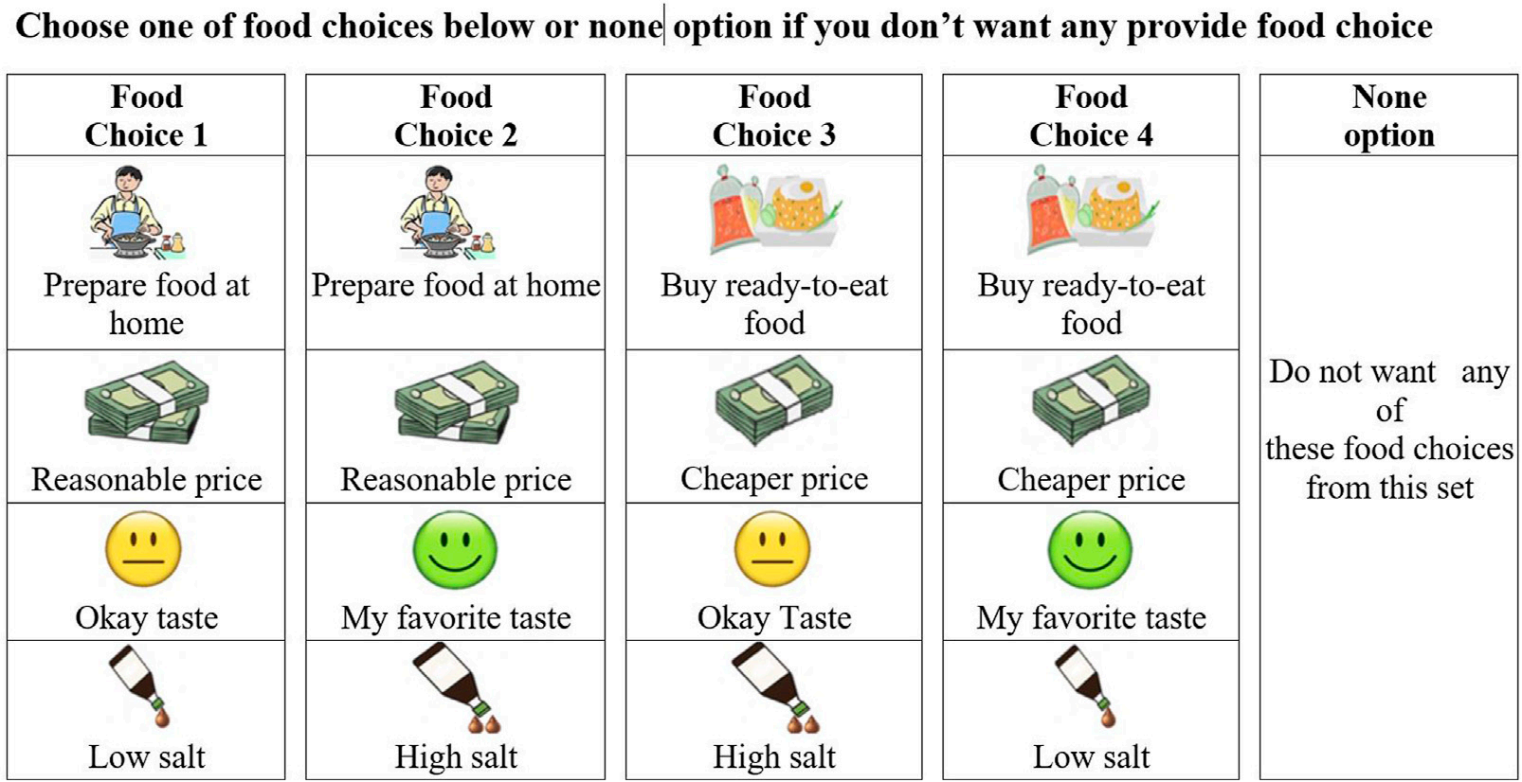
An example of a food choice set in a Discrete Choice Experiment (Chiang Mai, Thailand, 2019).

In addition to the four options in each choice set, a none option was also included. If subjects are unsatisfied with any of the available options, they may choose the opt-out option. By including an opt-out option, this prevents subjects from being forced to make a choice. As forcing subjects to make a choice induces bias, as they may not always make that same choice in real life [34]. An example choice set from the DCE is presented in Figure 1.

### Attribute Selection and Attribute Levels

Various attributes can influence consumer choices and should be considered in a DCE; however, some attributes may need to be excluded so that the choice sets are appropriate for the subjects’ cognitive ability [35]. As the number of attributes and levels increase, the number of possible choice sets increase, which can be overwhelming for subjects [24], potentially creating cognitive overload. This study considered a limited number of attributes and levels for each attribute to keep the complexity of the DCE simple.

Based on the literature, the major drivers of food choices are perceptions of convenience, cost, taste, and nutritional values [12, 30, 36, 37]. Convenience refers to the ease of physical access to food sources and the time spent buying and preparing food [36, 37]. Ready-to-eat foods require less cooking time, energy, and space, and therefore are considered more convenient than cooking food at home [17]. Food costs, such as food prices and the relative affordability of alternative food patterns [36, 38] also strongly influence food choices. Food taste is not only the taste of the food but also the aroma, texture, and pleasure response to foods [36]. Preferences for certain tastes, such as salty tastes, can strongly affect eating habits and are highly associated with hypertension [39].

**FIGURE 2:**
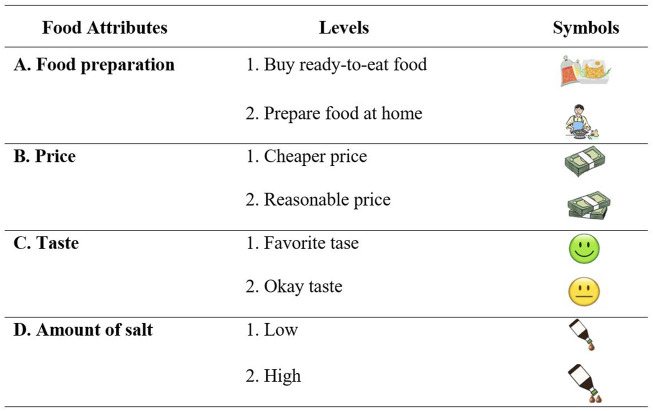
Food attributes for Discrete Choice Experiment (Chiang Mai, Thailand, 2019).

The attribute and attribute levels were carefully selected after several extensive discussions among a multi-disciplinary team of experts, consisting of health economists, epidemiologists, physicians, and public health experts. To ensure theoretical validity and cultural appropriateness, the research team pre-tested each of the DCE choice sets and the symbols representing the attributes with college students and staff at Chiang Mai University. In this study, four food attributes each with two levels were selected: convenience (preparing food at home and buying ready-to-eat food), price (reasonable price and cheaper price), taste (favorite taste and okay taste), and the amount of salt (low and high). The levels of these attributes were chosen to be qualitative as subjects may have a hard time quantifying exact values for some of these attributes, such as the amount of salt in milligrams or a taste test scale. The four attributes, levels, and symbols used in the DCE are presented in Figure 2.

Furthermore, several nutritional values could also influence an individual’s food choice and are associated with hypertension, e.g., saturated fat, trans fat, sugar, and salt. The amount of salt was specifically selected as an attribute because it has been estimated that dietary sodium consumption in Thai adults is nearly twice as high as recommended levels [40] and significantly elevated in rural northern Thailand [5, 6]. Specifically, sodium sources are predominately from the use of flavor enhancers such as fish sauce, soybean sauce, monosodium glutamate, and table salt added during consumption [6, 41] or cooking preparation [6, 10].

### Data Collection

The target population for this study was rural northern Thailand, particularly Chiang Mai province with the largest population in the northern region. Within Chiang Mai province, Bang Klang Subdistrict was chosen as it is located in the rural area of San Pa Tong District, not too far from the Muang District (City of Chiang Mai), where the research team was stationed, it has a well-established community network, which is beneficial to obtain representative data and a reasonable sample size, and lastly, hypertension is the main health concern in this area as it is the leading cause of illness, followed by respiratory tract infections, diabetes mellitus, kidney disease, and musculoskeletal diseases. In the Bang Klang Subdistrict, five of the largest villages out of eleven were selected. Subjects were residents from these villages and over the age of 18. A minimum sample size of 356 subjects was calculated using Taro Yamane’s formula [42] [*n* = *N*/(1+Ne^2^), *n* = 3,257/(1+ (3,257 x 0.05^2^)], where *n* is the sample size; *N* is the largest sample population; *e* is the margin of error. In total, 407 Thai adults were sampled from the five villages: 1) Sieo Village (N = 1462), 2) Tong Fai Village (N = 722), 3) Phra Chao Thong Thip Village (N = 429), 4) Ton Kok Village (N = 390), and 5) Pa Sak Village (N = 254), of which 403 completed the survey questionnaire and received an incentive gift valued $1.00 (30 THB).

The paper-based survey questionnaire was created in the Thai language and pilot tested by volunteer college students prior to data collection. The English version of the survey questionnaire was created using the repeated forward-backward translation procedure to preserve the original meaning [43]. In June 2019, the research team conducted 20-min face-to-face, one-on-one interviews with each of the subjects in the Thai language at each of the participating village community centers. Before the DCE questions were presented to the subjects, the interviewer carefully explained the symbols in this study. Following this, the interviewer performed an assessment to ensure subjects clearly understood the meaning of the symbols before proceeding to the DCE questions. For every DCE question, the interviewer described the various combinations of attributes and the opt-out option to the subject until they had no further questions before asking them to select one option. The research study was reviewed and approved by an affiliated Institutional Review Board (#HSR-18-19-712, 21 June 2019). All subjects provided informed consent.

### Hypertension Measurement

Trained health care professionals collected each subject’s blood pressure (BP) twice, once before and another 5 minutes after the interview. The average systolic and diastolic BPs of each subject were calculated. The BP measurement criteria used in this study were the 2017 AHA/ACC guideline [44] for the prevention, detection, evaluation, and management of high blood pressure in adults. The criteria recommend a blood pressure management goal of less than or equal to 130/80, rather than 140/90, for adults with diagnosed hypertension or heart disease. This recommendation promotes healthy blood pressure among Asian populations, in which high BP can contribute to more adverse cardiovascular health outcomes than in Western populations [45]. Hypertension at the time of measurement was considered if a subject’s average systolic BP was greater than or equal to 130 mmHg or their average diastolic BP was greater than or equal to 80 mmHg; otherwise, subjects have normotension. In Table 1, approximately 58% of the subjects (*n* = 234) have hypertension, and 42% have normotension.

**TABLE 1:** Sociodemographic characteristics of subjects associated with hypertension (Chiang Mai, Thailand, 2019).

Characteristics	Overall	Normotensive Group^a^	Hypertensive Group^b^	*p-value*
Age (mean ± s.d.)	59.64 ± 11.93	59.18 ± 12.12	59.97 ± 11.78	0.003^c^**
Gender n (%)				
Male	108 (26.80%)	36 (21.30%)	72 (30.77%)	<0.001^d^***
Female	295 (73.20%)	133 (78.70%)	162 (69.23%)	
Elderly (y ≥ 60) n (%)				
years ≥60	222 (55.09%)	89 (52.66%)	133 (56.84%)	<0.001^d^***
years <60	181(44.91%)	80 (47.34%)	101 (43.16%)	
Education n (%)				
Junior high school degree or above	111 (27.54%)	45 (26.63%)	66 (28.21%)	0.118^d^
Less than junior high school degree	292 (72.46%)	124 (73.37%)	168 (71.79%)	
Individual Monthly Income				
<5,000 Thai Baths (<150 US Dollars)	242 (60.05%)	103 (60.95%)	139 (59.40%)	<0.001^d^***
≥5,000 That Baths (≥150 US Dollars)	161 (39.95%)	66 (39.05%)	95 (40.60%)	
Occupation				
Daily Labor	154 (38.21%)	65 (38.46%)	89 (38.03%)	<0.001^d^***
Business	83 (20.6%)	29 (17.16%)	54 (23.08%)	
Government officer	13 (3.23%)	10 (5.92%)	3 (1.28%)	
Private employee	1 (0.25%)	0 (0.00%)	1 (0.43%)	
Farmer	40 (9.93%)	17 (10.06%)	23 (9.83%)	
Unemployed	107 (26.55%)	44 (26.04%)	63 (26.92%)	
Other	5 (1.24%)	4 (2.37%)	1 (0.43%)	
High Blood Pressure in Family History n (%)				
No	217 (53.85%)	96 (56.80%)	121 (51.71%)	0.001^d^***
Yes	186 (46.15%)	73 (43.20%)	113 (48.29%)	
Total observation	403	169 (41.93%)	234 (58.07%)	

a Normotensive Group (NG): Systolic BP <130 and Diastolic BP <80.

b Hypertensive Group (HG): Systolic BP ≥130 or Diastolic BP ≥80.

c Two-Samples t-test for differences between normotensive and hypertensive groups.

d Chi-Square test for differences between normotensive and hypertensive groups.

*Statistically significant for *p* ≤ 0.05; ** statistically significant for *p* ≤ 0.01; *** statistically significant for *p* ≤ 0.001.

### Statistical Analysis

The results from the DCE were analyzed separately in normotensive and hypertensive groups using a Mixed Logit (MXL) model. Based on the BFD, four individual attributes (food preparation, price, taste, and amount of salt) and five interactions between two attributes (food preparation X taste, food preparation X amount of salt, price X taste, price X amount of salt, and taste X amount of salt) were estimated in the MXL model. Furthermore, an alternative specific constant (ASC) variable for the none option was included as a fixed effect in the model to examine the opt-out effect.

The relative importance of each attribute was estimated to compare the explanatory power of each attribute on food choices. The partial log-likelihood method was used to calculate the relative importance based on how much each attribute contributed to the overall log-likelihood in the choice model [46]. The reduced model log-likelihood values were calculated when one attribute and any of its estimable interactions were excluded from the full model. Then, the partial effect of each attribute was calculated as the change in the reduced model log-likelihood from the full model log-likelihood. The relative effect was calculated as the percent change in the log likelihood. Thus, attributes that subjects consider more important in their choices will contribute more to the relative effect and have higher relative importance. It is noted that the statistical analysis used in this study can be found in greater detail in our previous study [30].

Significance was measured at *p-value* ≤ 0.05; however, there were a couple of variables that were marginally significant with *p-value* slightly greater than 0.05 (0.05 < *p* ≤ 0.1) that are worth mentioning. The individual attributes in the DCE were coded using effects coding to ensure that the systematic observed utility effects are uncorrelated with the intercept [47].

## Results

The sociodemographic characteristics of the subjects are presented in Table 1. The average age of the subjects was approximately 60 years old. About 73% were female, and 27% were male. Most subjects were elderly (55%), had an education level less than junior high school (72.46%), earned less than 5,000 Thai Bahts per month (∼<$150/month) (60.5%), and were daily laborers or unemployed (64.76%). More than half of the subjects had hypertension (58.07%) and reported having at least one family member with hypertension (53.85%). Among the sociodemographic characteristics, there were statistically significant differences between the NG and HG, except for the education characteristic.

The results from the Mixed Logit models for the DCE are presented in Table 2. Subjects in both the NG and HG were less likely to purchase ready-to-eat food (NG: β = –1.981; *p* < 0.001; HG: β = –1.807; *p* < 0.001). In other words, they preferred to prepare food at home. Furthermore, subjects in both the NG and HG were less likely to purchase food high in salt (NG: β = –1.931; *p* < 0.001; HG: β = –2.096; *p* < 0.001).

**TABLE 2:** Estimation of the mixed logit models (Chiang Mai, Thailand 2019).

Food attributes		Normotensive Group^a^				Hypertensive Group^b^			
		Estimate ( β)		Std.error	*p-value*	Estimate ( β)		Std.error	*p-value*
Individual effects									
Food preparation	Buy ready-to-eat food	−1.981	***	0.345	<0.001	−1.807	***	0.256	<0.001
	Prepare food at home (References)								
Price	Cheaper price	−0.066		0.109	0.545	−0.092		0.095	0.336
	Reasonable price (References)								
Taste	My favorite taste	−0.152		0.115	0.302	0.118		0.098	0.255
	Okay taste (References)								
Amount of salt	High salt	−1.931	***	0.269	<0.001	−2.096	***	0.251	<0.001
	Low salt (References)								
Interaction effects between attributes									
Ready-to-eat food X Favorite taste		0.078		0.114	0.491	−0.171		0.100	0.089
Ready-to-eat food X High salt		0.076		0.126	0.545	0.109		0.107	0.306
Cheaper price X Favorite taste		−0.128		0.083	0.121	−0.161	**	0.081	0.047
Cheaper price X High salt		0.215		0.080	0.788	0.157	**	0.078	0.043
Favorite taste X High salt		−0.251	**	0.123	0.040	0.052		0.104	0.619
ASC for opt-out (none option)		−1.016	**	0.499	0.042	−0.620	**	0.310	0.050
N		169				234			
Log-likelihood		−440.86				−564.85			

a Normotensive Group (NG): Systolic BP <130 and Diastolic BP <80.

b Hypertensive Group (HG): Systolic BP ≥130 or Diastolic BP ≥80.

* Statistically significant for *p* ≤ 0.05; ** statistically significant for *p* ≤ 0.01; *** statistically significant for *p* ≤ 0.001.

The interactions between attributes in the NG and HG varied and are presented in the bottom portion of Table 2. In the NG, the interaction between favorite taste and high salt had a significant negative impact on subjects’ food choices ( β = –0.251; *p* = 0.040); NG subjects were less likely to purchase food high in salt despite their favorite taste. In the HG, two interactions between attributes had a significant negative impact on subjects’ food choices. HG subjects were less likely to purchase food that was cheaper priced and their favorite taste ( β = –0.161; *p* < 0.047). In other words, they preferred food with a reasonable price and okay taste. HG subjects were less likely to purchase ready-to-eat food despite their favorite taste ( β = –0.171; *p* < 0.089). Instead, they preferred to prepare food at home with okay taste. Moreover, in the HG, the interaction between cheaper price and high salt had a significant positive impact on subjects’ food choices; HG subjects were more likely to purchase cheaper food despite high salt ( β = 0.157; *p* < 0.043). Hence, in foods that contain high salt, the subjects in the HG and NG made different trade-offs in their food choices (H1). Lastly, the estimate of the ASC variable for the *none* option indicated subjects in both the NG and HG groups were more likely to purchase food rather than opt out (NG: β = –1.061; *p* < 0.042; HG: β = –0.620; *p* < 0.05).

Table 3 presents the relative importance of the four attributes for the normotensive and hypertensive groups. The attribute, amount of salt, accounted for more than 50% of the log-likelihood in both the NG (50.55%) and HG (54.52%). Hence, the amount of salt significantly affected the subject’s food choices in both groups and thus had the highest relative importance in both NG and HG (H2). Further, the attribute, food preparation, added to 47.14% of the log-likelihood in the NG and 43.09% of the log-likelihood in the HG. The relative importance of the NG and HG differed in price and taste. Among the subjects in the HG, price had the third-highest relative importance (1.21%), and taste was the least relative important attribute (1.19%). However, taste (1.53%) and price (0.78%) were the third and fourth relative important attributes among subjects in the NG. Overall, subjects in the HG and NG had different food choices (H3).

**TABLE 3:** Estimation of the relative importance of food attributes (Chiang Mai, Thailand, 2019).

Food attributes	Normotensive Group a				Hypertensive Group b			
	Log-likelihood	Partial effect	Relative effect (%)	Relative importance	Log-likelihood	Partial effect	Relative effect (%)	Relative importance
Full model	−440.86				−564.86			
Food Preparation	−694.91	−254.05	47.14	2	−907.53	−342.68	43.09	2
Price	−445.07	−4.21	0.78	4	−574.44	−9.58	1.21	3
Taste	−449.13	−8.27	1.53	3	−574.32	−9.46	1.19	4
Amount of salt	−731.28	−272.42	50.55	1	−998.46	−433.61	54.52	1

a Normotensive Group: Systolic BP <130 and Diastolic BP <80.

b Hypertensive Group: Systolic BP ≥ 130 or Diastolic BP ≥80.

## Discussion

This study used a DCE survey method to examine food choices associated with hypertension among people living in the rural northern region of Thailand. With the unique design of a DCE, the study quantified the relative importance of the food attributes and interaction effects between the food attributes. The amount of salt was considered the most important attribute in both normotensive and hypertensive groups, accounting for more than 50% of the relative effect. Both groups of subjects in our study were likely to purchase low-salt food, which may infer that they had similar attitudes, beliefs, and knowledge about salt reduction. Based on the TPB, individuals with a greater awareness of the negative consequences of salt consumption, stronger personal and social norms, and higher salt-related knowledge, had an increased likelihood of purchasing low-salt food [5]. Even though our study found normotensive and hypertensive individuals valued the amount of salt as the most important attribute and were willing to purchase low-salt food, it is worth noting that the willingness to purchase may not be translated to the consumption. Studies suggest that both in Thailand, as well as in other countries, individuals generally have a reasonable knowledge of salt and its adverse effects in addition to favorable attitudes toward low-salt food; most individuals are less likely to take action to reduce salt consumption, and their blood pressure was still uncontrolled [6, 48–50].

Notably, when analyzing the interaction between the amount of salt and taste attributes, foods high in salt could also be confounded with an individual’s favorite taste. However, the interaction between these two attributes was not significant in the hypertensive group. In the normotensive group, subjects instead were less likely to purchase foods high in salt despite their favorite taste. The findings from the latter group are interesting as previous studies [51, 52] found that consumers typically choose taste over health and are not willing to accept poor taste in exchange for healthier foods. This counterintuitive finding may be explained from the distinction between hedonic choices (i.e., enjoyable and pleasurable foods) and utilitarian choices (i.e., functional and practical foods) [41]. According to Kahn et al. [51], consumers may be more inclined to choose foods with less salt if they predominately perceive the foods as utilitarian products. Therefore, their purchase decision is dominated by health rather than immediate pleasure. Whereas, when analyzing the interaction between the amount of salt and price attributes, subjects in the hypertension group were more likely to purchase foods high in salt at a cheaper price. Thus, it is hard to determine if their purchase decision is based on immediate pleasure or economic factors.

Food preparation was considered the second most important attribute in both normotensive and hypertensive groups. Both groups preferred cooking or preparing food at home over purchased ready-to-eat food. It is noted that due to the labor-intensive nature of Thai cooking [53], a food preparation at home has been decreased within urban Thai households. Instead, the high pressure to work longer hours and hurried lifestyles in urban cities have promoted ready-to-eat food [17, 54]. However, recent data suggest that ready-to-eat food consumption is relatively low among individuals residing in rural areas and the northern region of Thailand [55]. Specifically, people living in rural northern Thailand reported cooking or preparing food at home at least once in the past 3 months (98.40%); on average, they cooked or prepared food at home almost every day [6].

Regarding the sociodemographic characteristics, most of the subjects were married females. This supports the traditional culture of women’s role in relation to cooking for their families in rural Thailand [56]. In fact, most subjects were daily laborers or unemployed, and thus, their schedules may be more flexible with more time to make home-cooked food. The primary source of sodium intake among Thais, like many other Southeast Asian countries, comes from condiments added while cooking at home [57]. In rural northern Thailand, the main seasoning ingredients used when cooking are salt, followed by 1-2 teaspoons of monosodium glutamate (MSG) and seasoning cubes [6, 58].

The taste attribute was ranked third in the normotensive group and ranked the least important by the hypertensive group. While this finding is surprising, it may be explained by the socioeconomic status of the study sample that most of the subjects had an education level less than junior high school and were classified as low-income. The study on factors affecting food choices of older adults [59] found that higher-income individuals rated a meal that has a very good taste to be more important than did low-income individuals. The more educated individuals tended to rate lower price foods more important than the less-educated individuals.

In rural Thailand, extended families are common in which three generations often live together [60], and thus dietary habits are likely to be shared within the family [61]. Cooking, eating, and sharing a combination of dishes among family members and relatives are typical practices among Thais [62], leading to unawareness of overconsumption of sodium. Therefore, public health educational programs may pivot to consider cultural sensitivity focusing on daily habits. Individualized salt-reduction intervention programs should be aimed at the family level, such as identifying the traditional high-salt food the family often cooks, giving advice for low-salt cooking ideas for their traditional food, and monitoring daily food consumption.

### Limitations

There are some limitations to this study. The sample was small and lacking in demographic and socioeconomic diversity. A larger diverse sample could have provided more representation of the population. Furthermore, while DCEs closely reflect real-life decision-making in which people simultaneously make choices, each of which consists of several options, they are based on hypothetical choices as subjects do not actually choose the food, which may lead to hypothetical bias. As with any survey method, this limitation is common in DCE applications when choice tasks do not fully reflect reality in the characteristics of the choices [63]. In addition, the choice task of the DCE may become too cognitively challenging for subjects [64].

Moreover, since the interview was conducted at the village community center during midday, most of the sample were elderly females, as younger or male adults may have been at work during this time. This may have led to age-related factors (hearing, vision, memory loss) that could have affected the ability to obtain valid information [65], as well as age-related increases in blood pressure that could have influenced the proportion of hypertensive individuals in the sample. Future data collection should be gathered at various times throughout the day to obtain a more diverse range of ages and genders.

### Conclusion

This study aimed to contribute to the gap in the literature on understanding food choices associated with hypertension and determine the attributes that influence the food choices of rural northern Thais. The use of a DCE quantified the relative importance of attributes and the interactions between the attributes for normotensive and hypertensive groups. The most and second most important attributes in both groups were the amount of salt and food preparation at home, respectively. The interactions between the attributes revealed that normotensive people are more attentive to the amount of salt in their food than their hypertensive counterparts. The findings revealed that people in the rural areas of Thailand are aware of the amount of salt in their food choices and prefer home-cooked meals. As eating habits are concentrated at home and foods are commonly shared within the family, this study suggests that intervention programs for this population may benefit from focusing their attention on embracing low-salt cultural foods and providing guidance on low-salt cooking, especially for households with hypertensive members. Local health agencies and village health volunteers can play an essential role in educating, guiding, and training people in rural communities on how to add flavor without additional salt or reduce high sodium seasonings without losing flavor when cooking.
